# Electroconvulsive therapy (ECT) reduces suicidal behavior and suicide deaths: Response to Naismith et al.

**DOI:** 10.1017/S003329172510281X

**Published:** 2025-12-04

**Authors:** Charles H. Kellner, Randall T. Espinoza, Predrag Gligorovic, Alexander Sartorius

**Affiliations:** 1 Department of Psychiatry and Behavioral Sciences, Medical University of South Carolina, Charleston, USA; 2 Department of Psychiatry and Behavioral Sciences, University of Washington School of Medicine, Seattle, USA; 3 Psychiatry and Behavioral Medicine, Wake Forest University School of Medicine, Winston-Salem, USA; 4 Department of Psychiatry and Psychotherapy, Central Institute of Mental Health, Medical Faculty Mannheim, University of Heidelberg, Mannheim, Germany

We read with great interest the article titled ‘Systematic review and meta-analysis of the effectiveness of ECT in reducing suicidal ideation, self-harm, suicide, and mortality’ (Naismith et al., 2025) in *Psychological Medicine.* Suicide remains an urgent public health issue, and electroconvulsive therapy (ECT) is a vital treatment for our most severely ill patients, many of whom are acutely suicidal or express suicidal ideation. We wish to continue the discussion by highlighting other relevant literature, as well as to comment on aspects of the methodology of the review.

Naismith et al. limited their review to studies with both an ECT arm and a comparator arm. This choice omits relevant studies in which an acute course of ECT is administered and suicidal thoughts and behaviors are measured before and after the intervention. For example, the CORE study (Kellner et al., 2005) assessed the incidence, severity, and course of expressed suicidal intent in depressed patients who were treated with bilateral ECT. Suicidal intent was scored at baseline and before each ECT session with item 3 (score range 0 [absent] to 4 [attempts]) on the 24-item Hamilton Depression Rating Scale in 444 patients with unipolar depression. One hundred thirty-one (29.5%) patients reported suicidal thoughts and acts (score of 3 or 4) at baseline. After 1 week (three ECT sessions) scores decreased to 0 in 38.2%, after 2 weeks (six ECT sessions) in 61.1%, and at the end of treatment in 80.9% of the patients. Another large study, (Sienaert et al., 2022), from Swedish registry data, using the suicidal ideation (SI) item from the Montgomery-Asberg Depression Rating Scale-Self Assessment, reported that of the 1178 patients with pre-treatment suicidal ideation, 75.64% (N = 891) exhibited no SI at the end of treatment. Clearly, ECT has been shown to rapidly relieve expressed suicidal ideation/intent in depressed patients.

While Naismith et al. included 17 studies in their systematic review, their meta-analysis included only a subset of these: for all-cause mortality (seven), for nonsuicide mortality (two), and for suicide mortality (six). This was presumably because the other studies did not meet their stated criteria: ‘not every study reported the number of events for all three outcomes (all-cause, suicide, and non-suicide mortality), and we excluded studies where the denominator was not reported or was unclear (as for Babigian & Guttmacher, 1984).’ It is not clear to us why all three outcomes would be needed to include a study for which one or two of the relevant outcomes were measured. For example, the study by Rhee et al. (2021) reported decreases in all-cause and suicide-related mortality in a very large and well-matched geriatric sample, but was omitted from the meta-analyses, potentially altering the results.

Furthermore, a recent large-scale systematic review and meta-analysis of mortality associated with depression published in *World Psychiatry* (Chan et al., 2025) showed a dramatically reduced risk of death by suicide in patients treated with ECT versus those who were not (RR: 0.67, 95% CI [0.53–0.85]). Thus, a study addressing a very similar research question, and which additionally explored aggravating and attenuating factors, as well as sources of heterogeneity through subgroup and meta-regression analyses, came to a very different conclusion, showing ECT to be unequivocally protective.

We question a conclusion by Naismith et al. that ‘it is possible that ECT has no effect on suicide mortality’. As shown, data from other studies compellingly demonstrate a benefit from ECT on all-cause and suicide mortality. Their statement appears an unnecessarily negative assessment when considering the totality of clinical evidence. Taken out of context, such a statement may unwittingly play into the hands of anti-ECT and antipsychiatry forces. The recent disparagement of ECT in the WHO/United Nations publication, *Mental health, human rights and legislation: guidance and practice* (WHO, 2023) based on ideology, and the evidence-based rebuttal by ECT psychiatrists (Cooper et al., 2025) is a good example of the stigma that still plagues ECT. Our assessment of the entire evidence base on the effect of ECT on suicide is a much more salutary one than that of Naismith et al., and one that should encourage more frequent and earlier use of ECT by clinicians caring for severely depressed patients who are suicidal.
